# Recent Advances in the Development of Bioreactors for Manufacturing of Adoptive Cell Immunotherapies

**DOI:** 10.3390/bioengineering9120808

**Published:** 2022-12-15

**Authors:** Irina Ganeeva, Ekaterina Zmievskaya, Aygul Valiullina, Anna Kudriaeva, Regina Miftakhova, Alexey Rybalov, Emil Bulatov

**Affiliations:** 1 Institute of Fundamental Medicine and Biology, Kazan Federal University, Kazan 420008, Russia; 2 Shemyakin-Ovchinnikov Institute of Bioorganic Chemistry, Russian Academy of Sciences, Moscow 117997, Russia; 3 Izvarino Pharma, Moscow 108817, Russia

**Keywords:** adoptive cell immunotherapy, CAR-T, T cell, bioreactor, point-of-care

## Abstract

Harnessing the human immune system as a foundation for therapeutic technologies capable of recognizing and killing tumor cells has been the central objective of anti-cancer immunotherapy. In recent years, there has been an increasing interest in improving the effectiveness and accessibility of this technology to make it widely applicable for adoptive cell therapies (ACTs) such as chimeric antigen receptor T (CAR-T) cells, tumor infiltrating lymphocytes (TILs), dendritic cells (DCs), natural killer (NK) cells, and many other. Automated, scalable, cost-effective, and GMP-compliant bioreactors for production of ACTs are urgently needed. The primary efforts in the field of GMP bioreactors development are focused on closed and fully automated point-of-care (POC) systems. However, their clinical and industrial application has not yet reached full potential, as there are numerous obstacles associated with delicate balancing of the complex and often unpredictable cell biology with the need for precision and full process control. Here we provide a brief overview of the existing and most advanced systems for ACT manufacturing, including cell culture bags, G-Rex flasks, and bioreactors (rocking motion, stirred-flask, stirred-tank, hollow-fiber), as well as semi- and fully-automated closed bioreactor systems.

## 1. Introduction

A wide range of immunotherapeutic products have found extensive use in modern science and medicine. From various cell products to antibodies and cytokines, they are produced worldwide on an industrial scale. Therefore, scaling up of the biotechnological processes to meet the growing needs represents a serious challenge. Multiple optimization approaches exist for scaling up the production of immunotherapeutic products. The main principles are based on closed systems and automation. The more autonomous the process becomes, the cheaper and more efficient it gets for large-scale manufacturing. The use of closed systems can significantly reduce the risk of contamination, thereby increasing the quality of the product and lowering the infrastructural requirements.

The production of most immunological products is based on the patient’s own immune cells. Therefore, any biotechnological process related to cell cultivation, when scaled up and automated, will have to end up with bioreactor systems of some kind. In this regard, given the wide range of practical issues to be addressed, the development of modern bioreactors for industrial production of immunotherapeutics is extremely important. Industrial use of cell culture bioreactors is carried out after careful development and optimization of the protocol, since methodology and used materials can have a significant impact on the efficiency of the whole process [1].

**Figure 1:**
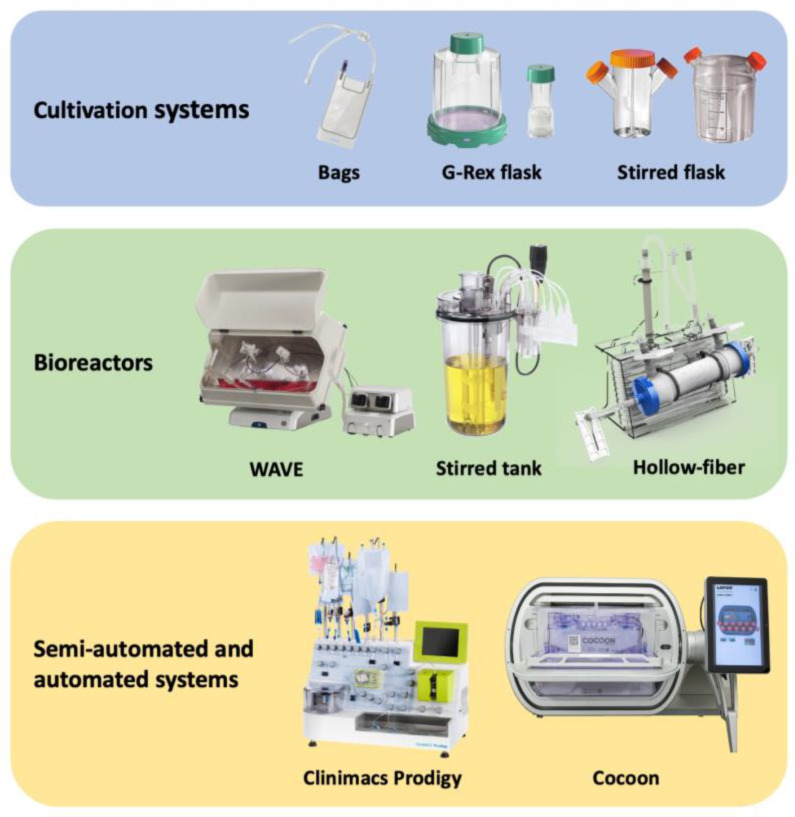
Primary types of cell cultivation systems, bioreactors, and automated systems. Cell cultivation systems are simple, require additional equipment, and involve manual cell processing at all steps. Bioreactors are more mechanized and allow certain steps to be completed in a closed aseptic mode. Semi-automated and automated systems are highly autonomous and require minimal human intervention.

Primary parameters to consider for biotechnology processes include pH, temperature, dissolved oxygen levels that can be controlled by medium oxygenation and replenishment (e.g., by perfusion), mixing of cell suspension to achieve uniform distribution of cells inside the bioreactor, etc. Bioreactor systems may use different basic principles and therefore may vary in performance and specific application. In addition, certain mechanical features may be advantageous for the efficient cultivation of one cell type while leading to low proliferation or even cell death in other cases. Thus, there is no universal solution to enhance throughput and achieve the required product quality, however, with careful selection of the appropriate system, high yields of quality cell products can be achieved. Below we describe various cultivation systems that are currently used in both academia and industry for the manufacturing of a wide range of cell products (such as CAR-T cells [2], Tregs [2], TILs [3], etc.). Technical solutions for cell product manufacturing can be classified into cultivation systems, bioreactors with a certain level of automation, and semi- and fully-automated systems (Figure 1).

## 2. Cell Culture Bags

Cell culture bags are made of polymeric materials and may have up to several aseptic ports for media input/output, sampling, and harvest. The main advantage of bags compared to T-flasks is a significantly lower risk of contamination. Bags may be manufactured from a wide range of gas-permeable polymers, such as silicone, ethyl vinyl acetate, polyolefins, etc. However, it should be taken into account that the bag material and its shape can significantly affect the cell proliferation and expansion.

Li et al. cultured human T cells in bags made of various materials such as silicone, polyolefin/ethyl vinyl acetate (EVA), fluorinated ethylene propylene (FEP) and compared results to regular T-flasks [4]. In polyolefin/EVA and FEP bags, cell expansion was nearly twice as slow as control T-flask, while the fold of expansion in silicone-made bags was the same as in the control. The authors assume that the higher gas permeability of silicone is the main reason for this observation.

Zuliani et al. performed the cultivation of tumor infiltrating lymphocytes in polyolefin bags and found that cell expansion was reduced by 9.8% compared to regular plates [3]. The authors then proposed a complex compartmentalized bag shape, which could provide local increase in cell concentration and thus provide better contact between TILs and feeder cells. Thereby, in principle, the bag shape may be re-designed to achieve enhanced cell growth for a given material.

The mixing option may also be implemented in bags by connecting input and output ports by a continuous tubing accompanied with a magnetic pump. Li et al. incubated human T cells in bags at different conditions: static, with periodic activation of magnetic pump, and with continuous pumping [3]. The use of the magnetic pump allowed it to avoid cell damage, unlike centrifugal or peristaltic pumps. The periodic pumping substantially increased T cell numbers, while the continuous pumping resulted in 75% decrease compared to the resting control T-flasks. Such results may be explained by the type of bag material and its effect on proliferation of T cells.

In general, the main advantages of the bags are a high level of aeration and relatively low cost. Bags of various intricate shapes can be designed for a local increase in intercellular interactions, which is important in the cultivation of lymphocytes. However, due to conflicting data for many types of immune cells, careful selection of bag material is required.

## 3. G-Rex Flask

One of the most popular devices for suspension cell culturing is the G-Rex flask, designed and manufactured by Wilson Wolf Manufacturing. G-Rex is a round cylindrical flask with a special gas-permeable silicone membrane at the bottom (Figure 1). The vessel is filled with media and then cells are inoculated. Cells sediment during cultivation and form a thick layer above the membrane. The large media volume provides sufficient quantities of nutrients while the silicone membrane ensures efficient gas exchange, which reduces the risk of oxygen starvation [5]. The media exchange is performed manually inside the laminar flow cabinet. Cells form a dense layer at the flask bottom, which makes it easy to replenish the medium without disturbing the cells. The indisputable advantages of the G-Rex system include low medium consumption and compatibility with standard laboratory equipment, such as laminar flow cabinets and CO_2_ incubators, which significantly reduces technical requirements for cell culture manipulations in comparison to conventional bags [6]. This significantly lowers the cost of switching from standard T-flasks to bioreactors, which facilitates the scale-up in laboratory, pre-clinical, and clinical settings (Table 1). G-Rex vessels are also more efficient than T-flasks and are better suited for the production of various cell products under GMP-compliant conditions [7,8,9].

Nevertheless, the G-Rex system has some drawbacks with regards to integration with automated GMP protocols for manufacturing of immunotherapeutic cell products, especially at the stages of cell seeding and quality control. The problems arise mainly due to the difficulty of transferring large volumes in a sealed and sterile manner from the flask to other devices, e.g., for washing and buffer exchange. The issues also include problems with sampling and limited maximal volume of the vessel. For example, for TILs manufacturing it may take up to 30 G-Rex flasks to produce the quantity of clinical grade cell product sufficient to effectively treat a single patient. At the same time, some types of perfusion bioreactors allow one-step production of large amounts of therapeutic cell products due to much higher volume of the expansion vessel. For example, in WAVE (Cytiva, Wilmington, NC, USA) rocking, the motion bioreactor volume of the cultivation bag can reach up to 100 L with a possible cell density of up to 10E7 cells/mL [10]. Eventually, using a large number of G-Rex vessels substantially complicates the quality control, making it necessary to individually analyze samples from each vessel for sterility, cell product identity, and efficacy.

Despite all the drawbacks, G-Rex remains one of the most popular solutions for scaling up biotechnological processes and is widely used in immunotherapy manufacturing. G-Rex is known for particularly efficient cell expansion when scaling up TILs production [6,11,12], which is explained by close interactions of lymphocytes with feeder cells followed by passive proliferation. Comparable efficacy TILs production was also demonstrated by gas-permeable bags (EXP-Pak by Charter Medical, Dublin, Ireland) [13]. For CAR-T cells, G-Rex flasks showed substantial efficiency. Gagliardi et al. demonstrated that the transduction efficiency of T cells in G-Rex compared to retronectin-coated bags was lower (55% versus 73%) [14]. However, the cell expansion and the total number of transduced effector cells was significantly higher in G-Rex than in the bags.

Overall, G-Rex flasks have proven to be an affordable and efficient solution for scaling up production without requirements for sophisticated additional equipment. In addition, G-Rex flasks consistently demonstrate high efficiency of cell cultivation due to the technology of medium oxygenation through a gas-permeable silicone membrane.

## 4. Rocking Motion Bioreactors

The main feature of the rocking motion bioreactors is a specialized swinging platform with heating elements and temperature sensors. Cells are grown in special plastic bags that are placed on this platform and are equipped with ports for gas injection, fresh medium infusion, waste medium removal, and sampling (Figure 1). Wave-like medium movements are generated inside the bag upon gentle platform rocking, allowing for agitation and aeration of the cell culture [15]. A sufficiently high cell density (up to 10E7 cells/mL) can be achieved in bioreactors of this type due to good aeration and constant inflow of fresh medium [10]. Cell culture bags can be supplied with a set of sensors (pH, DO) to monitor cell proliferation in real time, and specialized software can record changes in these parameters over time in the form of graphs. The bag volumes can vary considerably, allowing for a high degree of scalability of these bioreactors. For example, the working volume of bags for WAVE (Cytiva, Wilmington, NC, USA) ranges from 250 mL to as much as 100 L.

The rocking motion type of mixing can be both advantageous and disadvantageous depending on the specific experimental conditions (Table 1). Meng et al. used the WAVE bioreactor as a primary platform for incubating various types of immune cells and found that the expansion efficiency varied significantly [16]. This type of mixing had a positive effect on NK cells and dendritic cells; although, the expansion efficiency of cytokine-induced NK cells was comparable to the incubation under standard static conditions. Notably, WAVE bioreactor may also not be the most effective method for cultivating modified T cells, according to some reports. Somerville et al. expanded TILs and genetically modified peripheral blood lymphocytes (PBLs) in WAVE and static bags (3 L LifeCell culture bag (Baxter, Deerfield, MA, USA) in clinical grade settings [6]. TILs and PBLs had a similar fold of expansion in all cases, but the ratio of CD4+ and CD8+ T cells in the modified PBLs varied significantly. The numbers of effector CD8+ T cells in the WAVE were lower than in the static bag, which authors associate with unfavorable cell movement and media perfusion. The inter-cellular contact time is significantly reduced in this mode of cultivation due to rocking motion, which can negatively affect the expansion of individual subpopulations. Furthermore, the ratio of T cell subpopulations during the expansion may be affected by altered concentration of signaling molecules (e.g., cytokines, chemokines) and growth factors secreted by the cells. Furthermore, as a possible reason for the lower numbers of CD8+ T cells in WAVE bioreactors, the authors point at a potential toxicity of non-ionic surfactant Pluronic F68 used to reduce physical damage to cells during culture shaking.

In another study, Spanholtz et al. discovered that rocking motion has a clear positive impact on proliferation of natural killer cells. The NK cells were isolated from 16 donors and expanded in parallel in static Vuelife bags and WAVE bioreactor for 6 weeks. The average fold of expansion in bags was significantly lower than in WAVE at the end of the experiment. As a possible reason for this, authors indicate efficient media oxygenation achieved through rocking or waving of the cell suspension [17].

However, there is a growing body of evidence demonstrating the high efficiency of shaking and wave-like medium movements for TILs cultivation [18,19]. Sadeghi et al. showed that expansion of TILs and effector CD8+ T cells is significantly more rapid in WAVE than in classic bags [19].

Altogether, rocking-motion bioreactors represent the most common type of bioreactors used in cell product manufacturing. They have a high efficiency, can be connected to various sensors for monitoring the state of cell culture, but may have limited applicability for cells sensitive to intense medium flows. We believe that such bioreactors represent the optimal solution for clinical or large-scale production of immunological cell products. In comparison with G-Rex flasks, rocking motion bioreactors have a greater degree of automation, and therefore a lower risk of manufacturing human error and higher safety of the finished product.

## 5. Stirred Flasks and Stirred-Tank Bioreactors

The paddle mixing of cell culture medium was carried out in all stirred flasks and stirred-tank bioreactors. This type of mixing is associated with a high level of medium aeration, as well as a high shear stress of the cells. Stirred flasks are one of the oldest and most well-known cell culture reservoirs, consisting of a flask with a built-in mechanical or magnetic paddle stirrer [20]. Stirred-tank bioreactors are based on a similar mixing principle, but they are more autonomous and often equipped with sensors for monitoring pH and DO levels which altogether allows for better control over cell culture growth (Figure 1). Stirred-tank bioreactors may be capable of media replacement in a closed aseptic format, which helps to reduce the risk of contamination. The stirring mode is then turned off and cells are slowly sedimented to the bottom of the bioreactor within a few hours. The nutrient-exhausted media is then gently pumped out via the submerged tube and fresh media is added [21].

The positive effect of paddle mixing on the growth of various types of immune cells has been known for a long time. Examples include bone marrow mononuclear cells [22,23], NK cells [24], and many others. Fluid movements are essential for T cell cultivation, as T cells form clusters after activation and metabolism may be significantly disrupted within these clusters.

Carswell and Papoutsakis showed that paddle mixing has no negative effect on the cultivation of T cells at low speeds and that the use of medium additives that provide protection from mechanical stress can positively affect T cell expansion in stirred-tank bioreactors [25]. In addition, they also found that aeration of the culture medium by bubbling caused a rapid decrease in the rate of cell division, eventually leading to cell death and a reduction of total cell numbers.

Costariol et al. cultured T cells in the Ambr 250 (Sartorius, Göttingen, Germany) stirred-tank bioreactor with various blade rotation modes [26]. Two activated impeller blades rotated in opposite directions at speeds of up to 100–200 rpm. Control T cells were also cultured in a standard T-flask. The mode with two functioning blades had a poor effect on cell proliferation. The rotation of just one impeller, on the other hand, significantly enhanced the growth of T cells compared. The authors concluded that rotation of only one impeller at a speed of 200 rpm was optimal.

Foster et al. investigated the speed of paddle mixing on the expansion of cytomegalovirus-specific T cells using Bellco Glass stirred flask bioreactor and found that in this case expansion was more efficient compared to regular T-flasks [27]. They found that impeller speeds between 40 rpm and 75 rpm provided the highest cell growth, while rates beyond 75 rpm were harmful to cells.

Stirred flask and stirred-tank bioreactors are also used for manufacturing therapeutic cell products in clinical settings. According to Bohnenkamp et al., stirred flask bioreactors are useful for clinical scale expansion of T cells obtained from donors with cytomegalovirus infection [28]. In addition, stirred-tank bioreactors can be used in the production of viruses. Qu-Lai Tang et al. estimated the efficacy of lentiviral production in a 15 L stirred-tank bioreactor in comparison to T-flask and found that the lentiviral titer in the stirred-tank was 1.5 times higher [29].

Stirred-tank bioreactors are more commonly used for the production of viral vectors in HEK293 cells, or for the cultivation of mesenchymal stem cells (MSCs) [30]. Previously, this type of bioreactor was associated with low efficiency for immune cells. Some authors discuss the high sensitivity of T cells toward shear stress and the need for gentle handling [31]. However, recent reports suggest a high applicability of these bioreactors for T and CAR-T cell therapeutics. Thus, the application of stirred-tank bioreactors for T cell cultivation remains feasible and promising.

## 6. Hollow-Fiber Bioreactors

Hollow-fiber (HF) cartridges represent tubes filled with semi-permeable capillary fibrils and are constructed in such a manner that both intra- and extracapillary space can be utilized (Figure 1). The range of potential applications for HF cartridges in the pharmaceutical industry is extremely wide, including plasmapheresis, filtration of large volumes of liquids, and evaluation of changes in drug concentration over time. The most well-known manufacturers of hollow-fiber cartridges for biomedical applications are Repligen and FiberCell Systems. HF cartridges have been tested as cell proliferation chambers and demonstrated efficient expansion of adhesive cells, explained by a high inner surface area of the fibrils [32]. Interestingly, several studies also report the use of HFs for the expansion of suspension cells [33,34].

Cells can be grown both inside and in between the capillaries within the respective media circuit depending on the system configuration, cell type, membrane permeability, and required outcome [32]. One or both circuits can be connected to the medium tank via pump in various configurations to provide substance exchange, nutrients supply, and removal of metabolic byproducts by tangential filtration.

Most commonly, hollow-fiber bioreactors (HFBR) are used to produce various types of pharmaceutical protein products, such as antibodies [35]. Another application of HFBRs includes the production and collection of cytokines released during the activation and cultivation of T lymphocytes [36]. The convenience of HF-based systems is stipulated by a high ratio of the available surface area for cell adhesion and the efficiency of transmembrane nutrient exchange, resulting in greater cell density during cultivation. HFBRs can be highly adaptive and customizable due to the large number of monitored system parameters, such as media volume, membrane porosity, fibril diameter, their packing density, and the shape of their mutual arrangement inside the cartridge. To enhance the output of biological products, the fibrils can be increased in diameter and number, as well as being packed in a denser manner. As a result, extremely high concentrations of the final product can be achieved [33].

The suspension cultures, such as immune cells, are mostly grown in the extracapillary HFBR circuit in high densities (Table 1). The fresh medium is supplied through fibrils of the internal circuit, which minimizes the direct mechanical impact on the cells. The HFBR systems have long demonstrated their potential for scaling up the production of virus-transduced autologous T cells with sufficient efficiency and quality for further use in clinical settings [37]. In addition, due to their scalability, HFBRs may be useful in the industrial production of cell-based immunotherapeutics, particularly when large quantities of the cell product are required, for example, TILs [33].

The intracapillary compartment is rarely used for cultivation of suspension cells, however Nankervis et al. proposed using the Quantum automated system (Terumo) for expansion of T cells [38]. However, this bioreactor system is associated with significant drawbacks, such as the high likelihood of cell leakage into the system of tubes that provide medium exchange. The authors propose two solutions to this problem: vertical positioning of the cartridge and intracapillary media supply synchronized with simultaneous extracapillary waste removal.

The Quantum system has proven to be effective for cultivation of human immune cells [39,40]. Peripheral blood mononuclear cells grown in Quantum expanded 5 times faster than in control T-flasks [40]. Overall, despite being very modestly mentioned in the literature, the whole concept of Quantum system appears to be rather promising.

The main advantage of hollow-fiber bioreactors is flexible configuration that allows for the adjustment of cartridge structure, material, and membrane porosity. However, during cultivation with liquid flow in both compartments (as in the Quantum system [39]), very careful optimization of fluid velocities is required. Hollow-fiber bioreactors are not often used for growing immune cells, but they have high potential for further development.

## 7. Semi-Automated and Fully Automated Systems

The CliniMACS Prodigy (Miltenyi Biotec, Bergisch Gladbach, Germany) is the most widely used semi-automatic device for the manufacturing of various immunotherapeutic cell products (Figure 1). The range of available functions includes magnetic separation, cell cultivation and washing, and buffer formulation of the finished product. The CentriCult unit, a disposable plastic centrifuge capable of cell cultivation and pelleting, is the cornerstone component of the CliniMACS Prodigy [41]. The system is also equipped with a magnetic element to carry out immunomagnetic separation of specific cell populations, along with a disposable magnetic column and a set of tubes. Additional components include reagent stands, pinch valves for controlling fluid flow, peristaltic pump, liquid sensors, gas mixing unit, and a built-in inverted microscope for monitoring cell status during the cultivation [42].

CliniMACS Prodigy is probably the most convenient automated closed system available on the market, and it is frequently used for the purpose of clinical trials [42,43,44]. This approach presents several advantages, such as reduced infrastructural requirements to the manufacturing facility, full-cycle automation, reduced risk of contamination and human error, and GMP-compliant cell processing (Table 1). However, automated systems are costly and may not always be more efficient than less advanced methods. Granzin et al. found no differences in the expansion rate and cytokine production of NK cells grown manually and automatically [45].

Cocoon (Lonza, Basel, Switzerland) is another example of a closed automated system currently entering the market. This system, unlike CliniMACS Prodigy, does not allow cell separation or final product formulation, but it is capable of cell transfection, transduction, and expansion (Figure 1). In addition, Cocoon can link together several systems and perform parallel large-scale cell expansion with full electronic control over the process occurring simultaneously in several devices integrated within a single network [46]. This swarming approach may be found to be useful for industrial manufacturing of the off-the-shelf allogeneic cell products (Table 1).

CliniMACS Prodigy is probably the most popular system for automated manufacturing of adoptive cell immunotherapies. However, in the near future, it may encounter tight market competition with other new devices. Automated point-of-care (POC) systems represent the most advanced technological edge for manufacturing clinical-grade cell products. Only a few such systems are now available for clinical use, suggesting that more companies will be entering the competition very soon.

## 8. Conclusions

The rapidly expanding global market for all types of bioreactors for cell products makes it a highly lucrative field of science and technology that attracts major investments to accelerate the development of improved manufacturing practices. In recent years, the development of automated and semi-automated bioreactor systems has advanced rapidly to enhance the affordability, efficacy, and quality of various cell products. Expanding the scope of available technological solutions will allow for the manufacturing of a wide range of therapeutic cell products, including CAR-T cells, TILs, DCs, and NK cells (both autologous and allogeneic).

The fast-ever-growing field of immuno-oncology and customized cell therapies requires academic research and clinical facilities to accelerate the development to cover the unmet market needs [47]. However, the scaling up of the cell therapy industry is expected to result in a global manufacturing shortage that will mostly affect remote locations which are hard and costly to serve. We believe that the optimal solution to this problem is based on modular GMP-compliant bioprocessing units that will allow optimal allocation of resources and costs. The assembly of such units can be made fully automated to standardize and increase reproducibility of the whole process and reduce the user-associated risks.

Further advancements in both cell biology and hardware/software engineering will lead to more sophisticated closed automated POC platforms for rapid and cost-effective manufacturing of personalized cell immunotherapies. The POC systems are normally self-sustained and have lower infrastructural requirements which makes them more usable in various clinical settings. Such platforms are capable of regulating nutrient supply, medium exchange and waste removal. They also often include single-use disposable components and wireless sensors that monitor and record data in real-time.

The challenges that lie ahead of the scientific and biotech community, such as scaling up manufacturing of the cell products, automated monitoring, and sophisticated regulation of the whole process, will lead to the emergence of novel manufacturing platforms. Altogether, this eventually will transform the landscape from single institutions and small hospitals to an extensive and interconnected global network of manufacturing centers that will define the future of T cell therapeutics.

## Figures and Tables

**Table 1:** Main systems for adoptive cell therapy cultivation.

Culturing Method	Maximum Cell Density	Available Volume/ Size	Advantages	Disadvantages
Stirred Flask	1.7 × 10^6^ cells/mL	50 mL–35 L	Availability Scalability High level of aeration	Controversial efficacy data for the cultivation of some cell products Careful selection of the shape of blades is required
G-Rex flask	10–40 × 10^6^ cells/cm^2^	2 cm^2^–500 cm^2^ 8 mL–5 L	High density of cultivation Relatively low cost Can be operated using standard laboratory equipment (laminar flow hood, cell culture incubator)	Quality control check is required for each flask Additional steps needed for manual processing of the cell product Higher risk of contamination
Rocking motion bioreactors	10 × 10^6^ cells/mL	250 mL–100 L	Scalable to large volumes Uniform cell mixing High level of media aeration Perfusion capability Sensors for pH, dO, temperature Aseptic media addition and sampling	Low efficacy for some cell types Additional expensive equipment is required
Stirred tank bioreactors	2 × 10^6^ cells/mL	250 mL–1 × 10^4^ L	Great scalability Effective mixing with adjustable intensity High level of media aeration Range of sensors for monitoring cell culture Flexibility of control settings (rpm, shape of paddles, etc.)	Controversial efficacy data for the cultivation of some cell products Additional expensive equipment is required Careful selection of the shape of blades is required
Hollow fiber	1 × 10^9^ cells/mL	area (cm^2^) to volume (mL) ratio = approx. 100–200	Capability to culture cells at extremely high density Scalability Versatility (fibril configuration, cartridge size, etc.) Continuous addition of fresh medium and waste removal	Additional expensive equipment is required Need to carefully select the membrane/fibril properties (porosity, packing density, diameter, etc.) Complicated collection of the cell product
CliniMACS Prodigy	8 × 10^6^ cells/mL	500 mL	Semi-automated system Multicomponent device (all-in-one) Suitability for clinical application Availability of ready-made kits for various steps of the processes	High cost of the system and consumables Cell expansion is carried out in a centrifuge chamber (CentriCult Unit, a part of CliniMACS Prodigy system)

## Data Availability

Not applicable.

## Abbreviations

ACT	adoptive cell therapy
CAR-T cell	chimeric antigen receptor T cell
GMP	good manufacture practice
DC	dendritic cell
EVA	ethyl vinyl acetate
FEP	fluorinated ethylene propylene
HFBR	hollow-fiber bioreactor
MSCs	mesenchymal stem cells
NK	natural killer
PBL	peripheral blood lymphocyte
PoC	point-of-care
TIL	tumor infiltrating lymphocyte
